# Tumor-associated macrophages in tumor progression and the role of traditional Chinese medicine in regulating TAMs to enhance antitumor effects

**DOI:** 10.3389/fimmu.2022.1026898

**Published:** 2022-10-13

**Authors:** Jiatong Zhang, Jiafeng Gao, Jingwen Cui, Yongqiang Wang, Yipeng Jin, Di Zhang, Degui Lin, Jiahao Lin

**Affiliations:** ^1^ The Clinical Department, College of Veterinary Medicine, China Agricultural University, Beijing, China; ^2^ The Preventive Department, College of Veterinary Medicine, China Agricultural University, Beijing, China; ^3^ Center of Research and Innovation of Chinese Traditional Veterinary Medicine, China Agricultural University, Beijing, China

**Keywords:** tumor-associated macrophages, traditional Chinese medicine, cancer, immunotherapy, tumor microenvironment

## Abstract

**Purpose:**

To emphasize the importance of tumor-associated macrophages (TAMs) in tumor immunity and to describe the ways in which extracts from Traditional Chinese Medicine (TCM) achieve tumor therapy by modulating macrophages.

**Significance:**

By summarizing these available data, this review focused on TAMs and TCM and can build the foundation for future research on antitumor therapeutics.

**Methods:**

In this review, we summarized the key functions of TAMs in cancer development and overviewed literature on TCM targeting TAMs together with other immune cells aiming to enhance antitumor immunity.

**Conclusions:**

With an indispensable role in antitumor immunity, TAMs contribute to tumor progression, migration, invasion, angiogenesis, lymphangiogenesis, and immunosuppressive microenvironment. In recent years, TCM has gradually gained attention as a potential antitumor adjunctive therapy in preclinical and clinical trials. TCM is also a regulator of cytokine secretion and cell surface molecule expression in balancing the tumor microenvironment (TME), especially macrophage activation and polarization. Therefore, it is believed that TCM could serve as modifiers with immunomodulatory capability.

## Introduction

Macrophages are unique components of innate and adaptive immunity to defeat foreign pathogens and tumor cells (1). Tissue-resident macrophages spread through the blood and are usually immobile unless they are induced by stimulations (2). The initial state of tissue macrophages is called M0 macrophages, also known as Mφ macrophages, before being stimulated into the M1(classically activated state) or M2 phenotype (alternatively activated state). The phenotypes can be interchanged in response to various stimuli, (or activation) (3, 4) (**Table 1**). M1 macrophages are induced by Th1 cytokines, such as lipopolysaccharide (LPS), interferon-γ (IFN-γ), tumor necrosis factor α (TNF-α), granulocyte-macrophage colony-stimulating factor (GM-CSF) and glucocorticoid. They highly express major histocompatibility complex (MHC) molecules and produce nitric oxide (NO), reactive oxygen species (ROS), and pro-inflammatory cytokines, including interleukin (IL)-1β, IL-6, and IL-12. Above all, M1 macrophages are considered to exert antitumor activity.

**Table 1:** Classically and alternatively activated macrophages (3, 5).

	M1	M2a	M2b	M2c
Activators	LPS, IFN-γ, TNF-α	IL-4, IL-13	immune complexes, TLRs, or IL-1ra	IL-10, TGF-β, or glucocorticoids
Receptors	CD86, CD80, MHC II	CD163, CD206	CD86	CD163
Cytokines	TNF-α, IL-1, IL-6, IL-12, and IL-23	IL-10, TGF-β	TNF-α, IL-1, IL-6, IL-10	IL-10, TGF-β
Chemokines	CXCL10	CCL17, CCL13	CXCL13, CCL1, CCL20	
Arginase metabolism	L-citrulline and NO	polyamine and urea		
Functions	Th1 responses, tumor resistance	Th2 responses, type II inflammation, allergy	Th2 activation, immunoregulation	Inhibition of immune response, tissue remodeling

^1^ Characteristics of classically and alternatively activated macrophages. M1 macrophages are classically polarized macrophages, while M2 macrophages could be divided into M2a, M2b and M2c depending on different activators.

Initially, macrophages were simply divided into M1 and M2 two subtypes. With the later research, M2 macrophages could be divided into M2a, M2b, and M2c subtypes according to different activators. M2a macrophages are activated by IL-4 or IL-13, and M2b macrophages are stimulated by immune complexes. M2c macrophages are induced by IL-10 and transforming growth factor (TGF)-β. M2 macrophages secrete anti-inflammatory cytokines and chemokines, including a large amount of IL-10 and little of IL-12 as well as chemokine ligand (CCL)-17, CCL-18, CCL-22, vascular endothelial growth factor (VEGF), TGF-β, and Arginase1 (ARG1). Because of the different cytokines and chemokines they secreted, these three subtypes undertake different functions. M2a macrophages are responsible for Th2 responses, and M2b macrophages could also regulate immune status and therefore lead to the Th2 response. M2c macrophages could suppress immune responses and increase tissue remodeling (6). The dynamic character of phenotype allows macrophages to perform various functions. However, due to the complex internal and external environment, it is difficult to comprehensively summarize the types of macrophages by centralized classification. Therefore, when describing the subtypes of macrophages, it is favorable to choose a series of markers to replace the previous classification methods (3).

Tumor-associated macrophages (TAMs), which are exposed to the tumor microenvironment (TME), undergo M1-like or M2-like activation and then display tumor promoting or suppressing activities (7). When defining the classification of TAMs, we should analyze them in combination with the course of the tumor and the time of cell separation. Choose as many markers as possible, including surface proteins and intracellular proteomics (8), to jointly define the properties of macrophages. TAMs can express VEGF, TGF-β, angiogenesis chemokine CXCL12, and platelet-derived growth factor (PDGF), which promote the formation of partial blood vessels and lymphatic vessels of tumor and even further tumor invasion and migration. During tumor initiation, the infiltrated macrophages display the M1 phenotype, which secretes inflammatory cytokines to defeat tumor cells along with other immune cells (9). However, as cancer advances to the later stages, TAMs convert to the M2 phenotype and create an immunosuppressive microenvironment to support further cancer proliferation, invasion, and metastasis, leading to poor prognoses. However, it is difficult to classify them with specific markers, and a series of markers are typically used for classification (10). The biomarkers of tissue macrophages are complicated because of their distinct locations and functions. F4/80^hi^ cells have been identified as the phenotypic definition of tissue macrophages in mice. Additionally, human macrophages exhibit characteristics that are similar to those of mice macrophages (11).

M1 macrophages express CD68, CD86, CD80, and high MHC class II complex. Scavenger receptor (SR), mannose receptor (MR), low MHC class II complex, and ARG1 are used as M2 phenotype markers (5).

Traditional Chinese Medicine (TCM) has developed for many years and is used as an adjuvant to chemotherapy. Many antitumor natural products come from TCM. However, little is known about its underlying mechanisms and bioactivities because of the complex components and chemical structure and the difficult extraction and purification processes. In addition to the direct cytotoxic effects on tumors, TCM plays various immunomodulatory roles in TME, including angiogenesis inhibition, cell-cycle arrest or apoptosis induction (12), and immune cell regulators, such as activating antigen-presenting cells (APCs) and enhancing NK cell-mediated killing activity. Overall, TCM presents the ability to inhibit tumor progression, angiogenesis, invasion, and metastasis (13).

In this article, we summarized and discussed the characteristics and functions of TAMs in the TME and the mechanisms of TCM targeting TAMs in cancer biological therapy. The evaluations of TCM and TAMs will guide new opportunities in cancer therapeutic strategies.

## TAMs and tumor progression

TAMs play indispensable roles in tumor progression, including initiation, promotion, immune suppression, angiogenesis, invasion, and metastasis (14). In the early stages of the tumor, stromal cells secrete colony-stimulating factor (CSF)-1 and other factors to recruit macrophages, which are primarily antitumor M1-like macrophages. However, in the advanced stages, tumor cells secrete other anti-inflammatory cytokines and chemical factors, such as CCL-2 and epidermal growth factor (EGF), leading to the recruitment and conversion of TAMs from the M1 to the M2 phenotype. The flow cytometry results showed that macrophages from advanced stages of hepatic carcinoma were mostly MHC class II^low^ TAMs, which were alternatively activated (15). It has also been confirmed that macrophages could be induced from M1 to M2 in a direct or indirect contact co-culture system with tumor cells (16, 17).

Cancer-related inflammation (CRI) refers to the relevance between the instability of the genome and inflammatory mediators, which are mainly composed of TAMs and other white blood cells, representing a hallmark of cancer. The fact that inflammation induces tumor progression through endogenous and exogenous pathways, suggests a relationship between the initiation of cancer and chronic inflammation caused by inflammatory cytokines produced by TAMs (18).

DNA damage could destroy the stability of genome stability. Poor DNA repair, apoptosis disorder, and radiotherapy or chemotherapy can lead to tumor initiation (19). Oxygen-free radicals have also been found to be critical in the initiation and progression of tumors (20). TAMs could produce IL-1 and TNF-α, promoting the formation of oxygen free radicals and further stimulating the macrophage response to other agonists (21). Meanwhile, the accumulation of reactive oxygen species (ROS) encourages macrophages to differentiate into a pro-inflammatory state, and therefore participate in the inflammation-induced tumorigenesis (22).

The density of the infiltrated TAMs and the M2/M1 ratio increases as tumors develop, leading to a poor prognosis (23). For example, the high proportion of CD163^+^ tumor infiltrated macrophages is related to the poor clinical prognosis in clear cell renal cell carcinoma (RCC) (24), which is supported by another report where the decrease of macrophages partially inhibited the growth of hepatocellular carcinoma (HCC) (15). Clinical datasets show that the overall survival rate of patients with positive expression of M2 macrophages was significantly lower than patients with negative expression. Tian et al. (25) found that among patients with Wilms’ tumor, longer survival time is correlated with a lower density of M2 phenotype macrophages, suggesting that the M2 macrophage index could be a predictor in the pathological examination. CD11c/CD206 signature is associated with macrophage polarization and can be used as an index to predict the prognosis. A CD11c^high^/CD206^low^ immune profile leads to a favorable outcome (26). Moreover, TAMs with the M2 phenotype could even affect the efficacy of chemotherapy and radiotherapy through the suppression of T cells (27).

## TAMs in invasion and metastases

The protease produced or induced by invasive tumor cells can degrade the extracellular matrix (ECM). Thus, the invasion and migration of tumor cells are significantly enhanced compared to those of normal cells. In tumor stroma, TAMs produce enzymes, such as matrix metalloproteinases (MMPs) and urokinase fibrinolytic enzymes (uPA) to promote matrix degradation, and hence the invasion and metastasis of tumor cells (28).

As one of the MMPs, MMP-9 is a paracrine regulator of tumor progression (29) that degrades ECM, destructs the basement membrane, and spreads cancer through the circulatory system (30). The secretion of MMP-9 and VEGF by M2 phenotype TAMs was notably higher than that by M1 macrophages (31). It has been identified that MMPs are involved in the degrading and remodelling process of ECM (32), and induce epithelial-mesenchymal transition (EMT) by decomposing the adhesion molecules (33). EMT, a process that transits immotile cells to motile mesenchymal cells and therefore weakens the tight junction of tumor cells (34). Within these pathways, the transforming growth factor-β(TGF-β) is the primary regulator, which is also the key factor facilitating the proliferation and differentiation of TAMs (35). As demonstrated in gastric carcinoma (36) and hepatocellular carcinoma (37), EMT is related to the high infiltration of TAMs, which produce higher TGF-β1 than macrophages with other phenotypes.

## TAMs in angiogenesis and lymphangiogenesis

Inducing angiogenesis and lymphangiogenesis is one of the major characteristics of tumor cells, the symbol of tumor expansion to distant metastasis. TAMs regulate tumor angiogenesis and lymphangiogenesis in two approaches: paracrine and cell autonomous mode (38). As the tumor proliferates, the supply of oxygen becomes insufficient, generating a hypoxia tumor microenvironment. Macrophages are recruited to the regions between tumor and interstitial cells where vascularization is poor (39). After being stimulated by hypoxia-inducible factor (HIF-1α), TAMs release a set of angiogenic cytokines, such as vascular endothelial growth factors (VEGF)-A (40), TGF-β, CXCL12, PDGF, and MMPs (7), which in turn promote tumor angiogenesis (40). At the same time, macrophages deliver more VEGF-receptors (VEGFRs) under hypoxia to combine with VEGF in the TME, which affects downstream pathways and promotes the transformation of TAMs to M2 phenotype. TAMs could also activate endothelial cells in cervical carcinoma, which highly express VEGF-C and VEGF-D, and stimulate existing lymphatic endothelial cells’ proliferation (41). Furthermore, the existence of macrophage-derived lymphatic endothelial cell progenitors (M-LECP) has proved the autonomous mode. Under the stimulation of inflammatory factors, M-LECP could differentiate into lymphatic endothelial cells (LEC), contributing to pre-existing lymphatic vessels and subsequent lymphogenesis (38).

TAMs could overexpress HIF-1 and HIF-2, further up-regulating CXCL12. CXCL12 was found to be critical in enhancing the GM-CSF/Heparin-binding epidermal growth factor (HB-EGF) paracrine loop of colon cancer metastases in the liver, advancing tumor anti-apoptosis and the recruitment of TAMs (42). CXCL12 has also been identified to promote monocytes to differentiate into CD163^+^ macrophages and increase the expressions of VEGF and angiogenic chemokine CCL1 (43). To overcome the hypoxia and immunosuppress of the TME, a biomimetic nano-RBC system (V(Hb)) combined with hemoglobin–poly(ϵ-caprolactone) (Hb–PCL) and doxorubicin (V(Hb)@DOX)) was engineered. V(Hb)@DOX could effectively limit the recruitment of CD163^+^ M2-type macrophages and improve tumor hypoxia by reducing HIF-1α expression. Furthermore, the alleviation of the immunosuppressive TME decreased the secretion of MMP-9 and VEGF-A in tumors, which in turn inhibited tumor growth and metastasis (44).

## TAMs and immunosuppressive microenvironment

**Figure 1:**
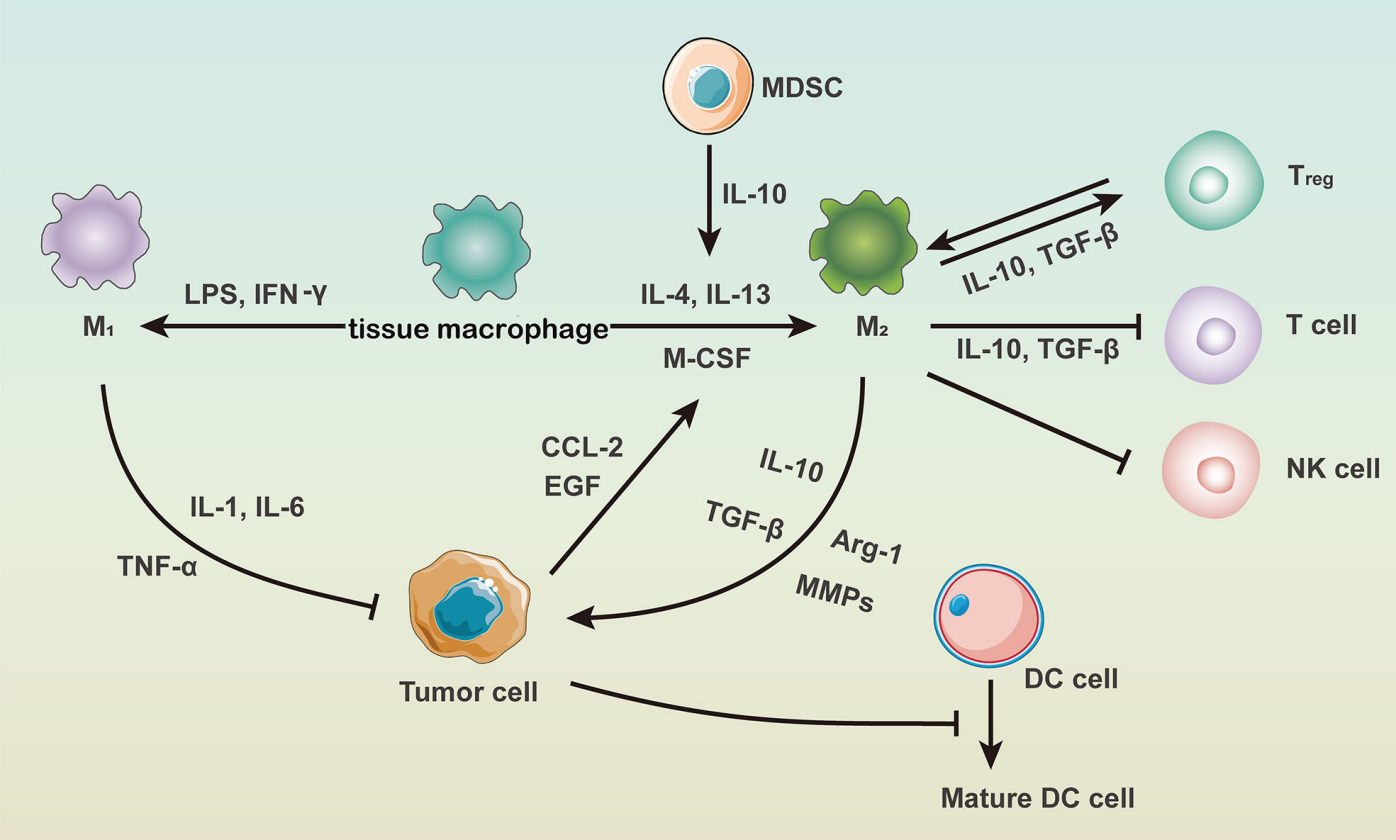
The role of macrophages in the immunosuppressive tumor microenvironment.

The immunosuppressive tumor microenvironment consists of tumor cells, endothelial cells, fibroblasts, ECM, and immune cells et al. Immune cells therein include macrophages, dendritic cells (DCs), T cells, B cells, myeloid-derived suppressor cells (MDSCs), natural killer (NK) cells and regulatory T cells (45). As chronic inflammation is essential in the immunosuppressive microenvironment, immune cells and inflammatory factors highly interacted with each other (46), which are summarized in **Figure 1**. It has been proposed that IL-10 secreted by MDSCs could down-regulate IL-12 produced by macrophages and thus induce macrophage polarization into the M2 phenotype (47).

TAMs restrain T cell-specific response in various aspects according to the recent findings. Extracellular vesicles (EVs), isolated from M2 phenotype macrophages, crippled CD8^+^ T cell proliferation, and killing activity, leading to tumor immune evasion in murine hepatocellular carcinoma (48), colon cancer (49) and gastric cancer (50). TAMs depletion with a nanocarrier named ^BLZ-945^SCNs/Pt, which delivers both CSF-1R inhibitor-BLZ-945 and platinum (Pt)-prodrug, can achieve the synergistic antitumor activity of chemoimmunotherapy. The decrease in TAMs significantly reduced the expressions of TAMs-derived VEGF-A and MMP-9 and further less lung metastasis, which demonstrated the therapeutic efficacy of targeting TAMs in tumors (51). Sun (52) reported that Doxorubicin (DOX)-loaded micelles with a hemoglobin crown (Hb-DOXM) have also achieved significant antitumor effects in reprograming the immunosuppressive microenvironment into the immunostimulatory microenvironment by augmenting the release of O_2_ and DOX and reducing the recruitment of M2-type macrophages in tumors.

For one thing, TAMs act as T cell activators using their surface MHC I or II molecules also by producing cytokines. For another, TAMs could induce T cell inhibition and exhaustion. Through direct and indirect regulations, TAMs are important in each of three steps to activate T cell response: specific binding of T cell receptors and MHC molecules on TAMs, costimulatory molecule signaling pathways, and environmental cytokines derived from TAMs (53). TAMs could secrete IL-10 to induce the expression of inhibitory receptors by T cells, such as programmed death (PD)-1 and cytotoxic T lymphocyte-associated antigen (CTLA)-4. The bindings of receptors and the corresponding ligands (PD-L1 and CD80/CD86) on the surface of TAMs, lead to negative regulations of T cell immune response, including apoptosis, anergy, and exhaustion (54).

Regulatory T cells can also weaken the immune functions of CD4^+^ and CD8^+^ T cells (55). Thymus-derived CD4^+^CD25^+^Foxp3^+^ regulatory T cells could increase the percentage of CD206^+^ and CD163^+^ macrophages differentiated from monocytes and up-regulate CCL18 and IL-1Ra produced by macrophages (56). Moreover, TAMs and DCs could increase the production of IL-10 and TGF-β, which transform naïve T cells into regulatory T cells (55) and further inhibit the antitumor immunity contributed by NK cells.

In summary, targeting the regulation of immune cell balance and augmenting tumor immunity in the microenvironment has always been the focus of tumor immunotherapy.

## TAMs in canine tumors

Similar to human tumors, many studies suggest that TAMs have a relationship with the grading of malignant tumors in veterinary science.

In canine lymphoma, tumor-infiltrating macrophages could be characterized as M1 and M2 according to iNOS, CD204 and CD163. As shown in the immunohistochemical results, the type of macrophages changed from M1 to M2 in the high histological grade. Among the two immunophenotypes of lymphomas, type B and type T, M2 macrophages have a dominant position in T-type lymphoma (57). Unlike human tumors, in many cases, it has been reported that CD204 is a better choice than using CD163 as the marker of the M2 macrophage subtype in canine tumors (58).

In canine mammary gland tumors, another result supported that tumor-infiltrating M2 macrophages have been correlated with the grading of malignant lesions (59). Furthermore, the high density of TAMs in canine mammary tumors has also been considered as a poor prognosis (60). TAMs also have a significant relationship with the expression of VEGF in canine mammary tumors, suggesting that TAMs synergistically promote tumor angiogenesis (61). Above all, TAMs may act as a potential target in the therapy of canine mammary tumors.

## TAMs regulated by TCM

Many studies supported that TCM plays an important role in cancer treatment, including promoting immune function, activating immune cells, enhancing the efficacy of antineoplastic, and reducing the side effects of radiotherapy and chemotherapy (62). Some kinds of TCM can directly inhibit the proliferation of tumor cells, while others suppress tumor growth, invasion, and metastasis indirectly by indirectly regulating the immune system (63). Vincristine and paclitaxel are common commercialized chemotherapeutics extracted from TCM in clinical applications. According to National Medical Products Administration, there are more extractions from TCM that are used as adjuvant therapies with radiotherapy and chemotherapy, such as lentinan, krestin et al. Since the 1980s, both China and Japan have approved the use of the mushroom polysaccharide, lentinan, as an adjuvant medicinal medication for the treatment of cancers. Lentinan was mainly used in treating lung (64), gastric, and colorectal cancers as adjuvant therapies and exhibited better efficacy and clinal response rates, as well as improved the quality of life of cancer patients, according to a review that summarized 9474 reported lentinan-associated cancer treatment cases (65). Additionally, polysaccharide-kureha (PSK), also known as Krestin, was authorized for the treatment of various cancers (66). PSK is frequently given orally, either alone or in combination with other drugs. Together with tegafur/uracil (UFT), PSK significantly increased stage II and stage III colorectal cancer patients’ 5-year disease-free survival and reduced the risk of recurrence and lung metastases (67).

The common active components from TCM with macrophage regulatory effects are glycosides, alkaloids, and polysaccharides, which could activate MAPKs, MyD88, and NF-κB related pathways by one or more receptors. The downstream phagocytic activity, ROS, NO, and relevant anti-tumor cytokines of TAMs are further enhanced accounting for the complete antitumor immune regulation (68).

### TCM activates antitumor phenotype and inhibits tumor-promoting phenotype of TAMs

TCM regulates macrophages in various ways, including activating anti-tumor macrophages, inhibiting the recruitment and activation of TAMs, transforming the phenotype of TAMs, and indirectly regulating TAMs by altering cytokine secretions in the tumor microenvironment.

It has been proven that acidic polysaccharides from Plantago major leaves could activate J774 macrophages and increase the release of NO and TNF-α (69). The polysaccharide extracts from Plantago depressa have also been shown as an immunomodulatory agent by promoting lymphocyte proliferation and NO production (70). Emodin inhibited the expressions of CCL2 and CSF1, which were involved in the differentiation of macrophages (71). It could also reduce the growth of EO771 and 4T1 breast tumor cells by suppressing macrophage migration and polarization, and inhibiting IRF4 and C/EBPβ signalings (72). Although astragalus polysaccharide (APS) could not inhibit the MCF-7 cell viability directly, it could activate RAW264.7 cells and up-regulate the production of NO and TNF-α, to induce the apoptosis of breast tumor cells (63). Ginseng polysaccharide (GPS) was shown to have similar functions as APS and exerted a cytotoxic effect against mice tumor cells *via* activating the peritoneal macrophages (PMs) rather than direct cytotoxicity (73).

Furthermore, TCM could cooperate with radiotherapy and chemotherapy, to enhance the curative effect of both and meanwhile alleviate the common side effects. A kind of water-extracted polysaccharides from Fuzi was found to promote the phagocytic activity and the release of NO, IL-6, IL-1, and TNF-α in RAW264.7 cells. Also, it had the ability to reverse the spleen index and thymus index in cyclophosphamide-induced immunosuppressed mice, demonstrating its possible application in antitumor therapy as an immunomodulator (74). Interestingly, not all TCM act as anti-tumor agents. Methanol extracts of Xanthium sibiricum roots (MXS) inhibit the NO, IL-6, IL-1β and TNF-α by suppressing IκBα and STAT3 signaling pathways in LPS-induced RAW264.7 macrophages (75).

Not only the monomers of TCM but also some complex TCM formulas have been proved to have macrophage-regulating functions. Bu-Fei Decoction (BFD), a conventional TCM constituted of six herbs, is often used for tonification and alleviating symptoms of lung cancer. In non-small cell lung cancer (NSCLC), BFD decreased the expressions of IL-10, PD-L1, and CD206 in TAMs induced *in vitro* by PMA and IL-4. Besides, BFD exhibited a dose-dependent inhibition of the invasion and migration of NSCLC cells *via* downregulating IL-10 and PD-L1 both *in vivo* and *in vitro* (76).

### TCM and macrophage polarization

M1-like TAM is the dominant phenotype suppressing tumor growth in the initial immune microenvironment of the tumor, but the M2 phenotype gradually replaces its leading position by recruiting tumor cells as the tumor advances to the later stages (7). In the tumor microenvironment, TAMs can switch between M1 and M2 states depending on the different signal inductions (13). Hence, finding new approaches to change TAMs from M2 to M1 phenotype could assist the antitumor immunity and prevent tumors from immune escape.

Astragalus polysaccharide (PG2) has been indicated as a modifier of macrophage polarization in NSCLC both *in vivo* and *in vitro*. PG2 enhanced the M1 polarization and reduced the CD206^+^ M2 cells in a dose-dependent manner. Also, PG2 could inhibit the tumor enhancement (including proliferation, clonogenicity to form tumorspheres, and invasion *via* IL-6/STAT3 signaling suppression) from a stem-cell-like phenotype of NSCLC induced by M2 macrophage. Furthermore, PG2 prominently strengthened the tumor-suppressive effect of cisplatin in NSCLC tumor-bearing mice models, but also alleviated dysuria and weight loss caused by cisplatin (77). Macrophages play an important role in baicalin-mediated inhibition of hepatocellular carcinoma (HCC). They were re-programmed towards the M1 phenotype to prevent tumor cells from immune escape, which is characterized as the descending proliferation and invasiveness of HCC cells. This repolarization was related to the autophagy-associated up-regulation of RelB/p52 (78).

A novel polysaccharide WCCP-N-b isolated from Cantharellus cibarius can induce M2-like bone marrow-derived macrophages (BMDMs), mouse peritoneal macrophages, and RAW264.7, to M1 phenotype. After being treated by WCCP-N-b, macrophages affected melanoma cell viability *via* increasing the production of TNF-α, which was cytotoxic to tumor cells (79). Water extract of ginseng and astragalus (WEGA) is reported to promote macrophages to express M1 markers and down-regulate M2 marker expressions simultaneously. Furthermore, WEGA also promoted immune responses, which were suppressed by cisplatin (62).

Emodin has received much attention due to its inhibiting effect on TAMs and its antitumor activity. It has been found that Emodin could inhibit IRF4, STAT6, and C/EBPβ signaling pathways *in vivo* to suppress macrophage infiltration and M2 polarization accompanied by T-cell activation, and therefore reduce breast cancer growth (72). Moreover, Emodin significantly inhibited breast cancer lung metastasis by inhibiting M2 polarization in metastatic lungs (80). Emodin inhibited the activation of NF-κB, STAT1, and IRF5 signaling pathways induced by LPS/IFNγ, and the stimulation of STAT6 and IRF4 signaling pathways stimulated by IL4 (81). Taken together, Emodin adopts an inhibitory effect on tumor growth by restoring macrophage homeostasis in the tumor-suppressive immune microenvironment.

### TCM and TME regulation

Some TCMs have direct cell killing effects, while most have lower cytotoxicity, but could enhance the bioactivity of immune cells or inhibit immunosuppressive cells for anti-tumor purposes.

Evidence showed that a polysaccharide extracted from the whole plant of *Plantago Asiatica* L. could recruit immune cells (DCs, macrophages, and T cells) in the murine breast tumor model and accelerate the maturation of DCs, which promoted the proliferation and differentiation of T cells. Plantain polysaccharides had no direct cytotoxicity to breast tumor cells. However, it inhibited tumor growth by promoting the autoimmune response in mice (82). Modified citrus pectin (MCP) has been identified to accelerate the activation of the T-lymphocyte subset, B cells, and NK cells (83). Astragalus polysaccharide (PG2) not only regulated the macrophage phenotype but also promoted the maturation of immature DCs and recruitment of CD8^+^ T cells for anticancer immune response in NSCLC (77).

Besides, many studies showed a close relationship between immunosuppressive cells and TCM. TCM could suppress the recruitment and metastasis of immunosuppressive cells when tumor-promoting immune cells are dominant in quantity and function. Silibinin, extracted from milk thistle, dwindled tumors in 4T1 tumor-bearing mice by decreasing MDSCs infiltration and M2-like polarization of macrophages. However, in an immunodeficient mouse model, similar efficacy was not observed, suggesting the anti-tumor response of Silibinin was based upon the integrity of the immune system (84). Maitake D (MD)-Fraction, a β-glucan extracted from Grifola frondose, inhibited the growth of mammary carcinoma and colonic adenocarcinoma cells and enhanced immune cell infiltration in the tumor microenvironment, including T cells, B cells, DCs and NK cells. DC maturation, specific T cell responses, and the infiltration and anergy of Tregs and MDSCs were induced by orally administered MD-Fraction, suggesting the significance of converting immunosuppressive elements of the TME in tumor immunotherapy (85).

Not only polysaccharides from TCM could enhance the immune system, but other types of natural products, including terpenes, alkaloids, saponins, and flavonoids, also have the ability. Andrographolide, an isoprenoid extracted from Andrographis paniculate, has been reported to exhibit cytotoxicity to nearly all kinds of cancer cells and mediation of the immune system (86). In another study, Andrographolide released a high level of IL-2 and IFN-γ, promoted cytotoxic T lymphocyte (CTL) production and prolonged the survival time of mice bearing lymphoma (87).

### TCM delivery system

Considering the instability and low bioavailability of active components from natural TCM, it is quite challenging to apply them directly *in vivo*. In recent years, the rapid development of nano-drug delivery systems has made it possible to deliver TCM or employ TCM as drug carriers for cancer treatment. A growing amount of TCM has been delivered to tumor tissues and their stroma through nano-drug delivery, such as liposome, or precision targeted therapy and immune regulation. Astragalus polysaccharide liposome (APSL) has been demonstrated to enhance the phagocytosis of murine peritoneal macrophages and speed up the DC-mediated immune reactions compared to applying AP alone (88). Moreover, cell-membrane-coated nanoparticles showed high efficiency in passing through the biofilm barrier and slowing down the metabolism of the loaded drugs. A novel macrophage-biomimetic drug delivery system carrying Saikosaponin D was reported to inhibit cell migration of MCF-7 and 4T1 cells *in vitro* and significantly reduced tumor growth and lung and spleen metastasis by promoting dephosphorylation of AKT and Erk in tumor-bearing mice (89).

### TCM and canine tumors

The application of TCM in human treatment has gradually increased because of its chemopreventive and chemotherapeutic effects. However, its role in the small animal clinical field should not be underestimated. TCM could also be considered as an approach for clinical therapy to inhibit the growth of canine tumor cells. For example, Paclitaxel has been widely used to treat lung, ovarian, and breast cancer. It was reported to inhibit the migration of canine hemangiosarcoma (HSA) cells with the increase of time and concentration (90). In canine melanoma cells, oral paclitaxel was also tested to decrease the proliferation of tumor cells both *in vivo* and *in vitro* by arresting cell cycle (91). Canine mammary tumors are common among female dogs and the risks of malignancy are relatively high. BmKn-2, a peptide extracted from the venom of scorpions, has been proved to have antitumor activity in both human and canine tumor cells. It inhibited canine mammary gland tumor cell proliferation *via* inducing apoptosis, which was represented by the decrease of Bcl-2 to Bax ratios (92). Besides the direct tumor-killing effect, TCM could contribute to the immunomodulatory effects targeting immune cells and consequently hinder tumor progress. Our team has been focused on the study of TCM in antitumor immunity regulations and proved that although Plantain polysaccharide (PLP) showed no cytotoxicity to canine mammary cells (CIPp), conditioned medium obtained from PLP to DCs had inhibitory effects on CIPp cells. Moreover, it could promote the maturation of DCs and thus facilitate the proliferation of lymphocytes, which exert the main toxicity effect (82). All in all, TCM is of great significance in regulating animals with poor immune status, especially for the tumor patients.

## Conclusions

Overall, TAMs could suppress anti-tumor immunity by promoting cancer proliferation, invasion, metastases, and angiogenesis. Besides, TAMs contribute to the immunosuppressive microenvironment to further advance cancer development. TCM has been proved to be an effective method for reprogramming TAMs and other immune cells and turning the immunosuppressive microenvironment into an antitumor one. In this study, we provide support for further studies on antitumor immunity and immunotherapy.

## Author contributions

Writing—original draft preparation, JZ and JC. Writing—review and editing, JL and JG. Working concept and design, YJ and YW. Data collation, DZ. Supervision, JL and DL. All authors contributed to the article and approved the submitted version.

## Funding

This work was supported by the National Nature Science Foundation of China (Grant no. 31972730), the 2115 Talent Development Program of China Agricultural University (Grant no. 00109023), and the Special Fund Project of Fundamental Scientific Research Business Expenses of China Agricultural University (Grant no. 2020TC009).

## Conflict of interest

The authors declare that the research was conducted in the absence of any commercial or financial relationships that could be construed as a potential conflict of interest.

## Publisher’s note

All claims expressed in this article are solely those of the authors and do not necessarily represent those of their affiliated organizations, or those of the publisher, the editors and the reviewers. Any product that may be evaluated in this article, or claim that may be made by its manufacturer, is not guaranteed or endorsed by the publisher.
